# Correction: Sczelecki, S.; Pitman, J.L. The Validation of a Precursor Lesion of Epithelial Ovarian Cancer in *Fancd2*-KO Mice. *Cancers* 2023, *15*, 2595

**DOI:** 10.3390/cancers17111869

**Published:** 2025-06-03

**Authors:** Sarah Sczelecki, Janet L. Pitman

**Affiliations:** The School of Biological Sciences, Te Herenga Waka Victoria University of Wellington, Wellington 6012, New Zealand; sarah.sczelecki@vuw.ac.nz

## Text Correction

There was an error in the original publication [1]. The Supplementary Materials did not include the extended methods as referenced in the main text [1].

A correction has been made to Supplementary Materials:

**Laser Capture Microdissection**

Prior to sectioning, the cryostat and all cutting surfaces were cleaned with RNase-free 100% ethanol to ensure an RNase-free working area. Tissues from both WT and KO samples were equilibrated to −20 °C for 30 min before sectioning, and PET membrane slides (MMI GmbH, Eching, Germany) were exposed to UV light for 30 min prior to use to improve adhesiveness. The frozen tissue was cut to produce approximately 6 × 10 µm serial sections on each PET membrane slide, using the slide mounting tool and warming behind the section to help adherence. Following the collection of the first slide, 200 µm was discarded between each slide and another set of six serial sections were collected as above on 3–4 additional slides and stored at −80 °C until required. Prior to laser capture microdissection, slides were thawed briefly at room temperature and processed in preparations of the following solutions: (i) 100% RNase-free ethanol for 30 s; (ii) 70% RNase-free ethanol for 15 s; (iii) RNase-free H_2_O for 45 s; (iv) Haematoxylin Gill 3 (Sigma-Aldrich, St. Louis, MO, USA) for 15 s; (v) RNase-free H_2_O for 30 s; (vi) 95% RNase-free ethanol for 30 s; and (vii) 100% RNase-free ethanol for 30 s. After staining, slides were air-dried to completion and then immediately microdissected. Three to six caps of tissue for each sample type were collected per biological replicate.

**Immunohistochemistry**

The slides were initially heat-treated at 37 °C for 2 h to ensure the adhesion of paraffin sections to the slides. The slides were then deparaffinized in two exchanges of 100% Xylene for 5 min each and rehydrated in preparations of the following series of alcohols: (i) 2 × 100% ethanol, (ii) 95% ethanol, (iii) 70% ethanol and (iv) 50% ethanol, all for 3 min. Endogenous peroxidase activity was blocked using a solution of 3% H_2_O_2_ in methanol (*v*:*v*) for 10 min at room temperature. The slides were then rinsed for 2 × 5 min in 1× PBS pH 7.4 in preparation for heat-induced epitope retrieval. Antigen retrieval was performed for 20 min at 95 °C in either sodium citrate buffer (10 mM sodium citrate, 0.05% Tween 20, pH 6.0) or Tris-EDTA buffer (10 mM Tris Base, 1mM EDTA solution, 0.05% Tween 20, pH 9.0) depending on the primary antibody used (Table S4). The slides were then rinsed for 2 × 5 min in 1× PBS pH 7.4 and blocked in 10% Normal Sheep Serum (NSS) for 1 h at room temperature. Blocking buffer was drained off the slides and primary antibodies were diluted in antibody diluent (0.5% NSS in 1× PBS pH 7.4), as detailed in Table S4, and applied to slides overnight at 4 °C. After the overnight incubation, slides were washed for 2 × 5 min in 1× PBS pH 7.4, and the secondary antibody diluted in antibody diluent (Table S4) was applied to the slides and left to incubate for 1 h at room temperature. Slides were washed again for 2 × 5 min in 1× PBS pH 7.4 before the addition of Diaminobenzidine (DAB) solution (0.05% DAB, 0.015% H_2_O_2_ in 1× PBS pH 7.4) and the colour was developed for 1–3 min depending on the antibody. The slides were washed for 2 × 5 min in 1× PBS pH 7.4 and counterstained with Haematoxylin Gill 3 (Sigma-Aldrich, St. Louis, MO, USA) for 2 min followed by bluing by rinsing in tap water for 12 min. Finally, the slides were dehydrated through 4 exchanges of alcohol (95%, 95%, 100%, and 100%) for 5 min each and then cleared through 3 changes of 100% Xylene and cover-slipped using Depex mounting media (BDH Biochemicals, London, UK).

**Multiplex GeXP Assay**
**Custom Design of a Multiplex GeXP Assay**

Using available online tools (IDT and Life Technologies Oligo Analyzers), all primers in each set were compared to ensure there were no strong intra- or inter-primer interactions, and if there were, primers were redesigned. A forward (5′-AGGTGACACTATAGAATA-3′) and reverse (5′-GTACGACT CACTATAGGGA-3′) universal primer sequence was added the 5′ end of each primer to obtain compatibility with the Genome GeXP Genetic Analysis System (Beckman Coulter, Fullerton, CA, USA). This universal sequence serves as a template for universal primers in the reaction mixtures for use in subsequent amplification steps to ensure equal amplification efficiencies across all genes in a set. All gene PCR products were initially validated in single-plex using whole mouse ovary cDNA as the template, confirming the functionality of the primer pair by the presence of only one expression peak and, therefore, PCR product.

**Reverse Transcription (RT)**

An RNA template was added to a pool of all reverse primers of varying, pre-optimised concentrations (SI Appendix, Tables S1–S3), reverse transcriptase and associated buffer (as per the manufacturer’s instructions, Beckman Coulter, Fullerton, CA, USA) in a 20 µL reaction volume. A Kanamycin RNA (KanR) exogenous positive control was also included, resulting in a consistent 325 np peak when samples were separated on the GeXP machine. The RT reactions were performed under the following conditions: 1 min at 48 °C, 60 min at 42 °C, and 5 min at 95 °C in a Corbett Rotorgene qPCR machine to avoid the well-to-well variation of a standard thermocycler. The no-template control contained one peak at 325 bp corresponding to the exogenous KanR control and the RT-negative control contained no peaks. In addition to the negative controls, a positive control of an RNA mixture derived from mouse ovary tissue and mouse ovarian cancer cell lines, where all genes are expressed, was included to ensure assay success.

**Polymerase Chain Reaction (PCR)**

The resulting RT sample was used as the template for the subsequent PCR. Therefore, 9.3 µL of the final RT reaction was mixed with all forward primers of each GeXP set to a concentration of 20 nmol, Thermo-Start DNA Polymerase (Thermo Fisher Scientific, Waltham, MA, USA) and associated GeXP buffer, in a 20 µL final reaction volume (as per the manufacturer’s instructions, Beckman Coulter, Fullerton, CA, USA). The PCR was again performed in a Corbett Rotorgene qPCR machine under the following conditions: 10 min at 95 °C, then 35 cycles of 30 s at 94 °C, 30 s at 55 °C and 1 min at 70 °C. PCRs were completed in technical duplicate.

**GeXP Sample Separation and Data Analysis**

Samples were prepared for analysis following the manufacturer’s instructions, which is briefly outlined here. The capillary array was pre-heated to 50 °C for 15 min prior to analysis, followed by 3 × 0.4 mL manifold purges and 3× capillary fill operations. For each sample, 6.15 µL of neat PCR product was added to 1.025 µL of DNA size standard—400 (Beckman Coulter, Fullerton, CA, USA) and 74.825 µL of SLS Buffer (Beckman Coulter, Fullerton, CA, USA). The resulting 82 µL sample was split into 2 × 40 µL samples in the GeXP sample plate with 30 µL of mineral oil overlaid to avoid sample evaporation. Therefore, for every RNA sample, there was a total of four technical replicates. PCR products were separated by the standard fragment analysis method (frag-3 protocol) on the GeXP. Peaks were manually associated with each gene based on size results from previous single-plex optimisation steps and relative fluorescent units were exported to Excel for subsequent analysis. Genes were normalised to the most moderately expressed endogenous control, *Ppia*, in each sample, and the resulting fold-changes of the four technical replicates were averaged. Then, the fold-changes of the biological replicates for each experimental group were averaged for graphical representation.

The authors apologise for any inconvenience caused and state that the scientific conclusions are unaffected. This correction was approved by the Academic Editor. The original publication has also been updated.

**Supplementary Materials:** The following supporting information can be downloaded at: https://www.mdpi.com/article/10.3390/cancers15092595/s1, Supplemental Methods; Figure S1: Immunohistochemistry of common epithelial ovarian cancer markers; Figure S2: LCM of mature GCs and sex cords; Figure S3: Unbiased analyses of relative gene expression data of sex cords and ovarian stroma; Figure S4: Volcano plots representing non-significant gene expression changes; Table S1: Genome GeXP Multiplex Assay Set 1; Table S2: Genome GeXP Multiplex Assay Set 2; Table S3: Genome GeXP Multiplex Assay Set 3; Table S4: Detailed information about antibodies used for immunohistochemistry; Table S5: Summary of staining intensity of immunohistochemical markers in representative samples; Table S6: Summary of all pairwise comparisons made for Gene Set Enrichment Analysis and the resulting number of enriched gene ontology (GO) terms for each comparison.
