# Supplementary material for: The Validation of a Precursor Lesion of Epithelial Ovarian Cancer in Fancd2-KO Mice
Source: Cancers (Basel). 2023 May 3;15(9):2595. doi: 10.3390/cancers15092595 (PMC10177028; doi:10.3390/cancers15092595)
Supplement: Supplementary file 1 [file cancers-15-02595-s001.zip › cancers-2314115-supplementary.pdf]

Supplemental Methods

Laser Capture Microdissection

Prior to sectioning, the cryostat and all cutting surfaces were cleaned with RNase-free 100% ethanol to ensure an RNase-free working area. Tissues from both WT and KO samples were equilibrated to −20 °C for 30 min before sectioning, and PET membrane slides (MMI GmbH, Eching, Germany) were exposed to UV light for 30 min prior to use to improve adhesiveness. The frozen tissue was cut to produce approximately 6 × 10 μm serial sections on each PET membrane slide, using the slide mounting tool and warming behind the section to help adherence. Following the collection of the first slide, 200 μm was discarded between each slide and another set of six serial sections were collected as above on 3–4 additional slides and stored at −80 °C until required. Prior to laser capture microdissection, slides were thawed briefly at room temperature and processed in preparations of the following solutions: (i) 100% RNase-free ethanol for 30 s; (ii) 70% RNase-free ethanol for 15 s; (iii) RNase-free H_2O for 45 s; (iv) Haematoxylin Gill 3 (Sigma-Aldrich, St. Louis, MO, USA) for 15 s; (v) RNase-free H_2O for 30 s; (vi) 95% RNase-free ethanol for 30 s; and (vii) 100% RNase-free ethanol for 30 s. After staining, slides were air-dried to completion and then immediately microdissected. Three to six caps of tissue for each sample type were collected per biological replicate.

Immunohistochemistry

The slides were initially heat-treated at 37 °C for 2 h to ensure the adhesion of paraffin sections to the slides. The slides were then deparaffinized in two exchanges of 100% Xylene for 5 min each and rehydrated in preparations of the following series of alcohols: (i) 2 × 100% ethanol, (ii) 95% ethanol, (iii) 70% ethanol and (iv) 50% ethanol, all for 3 min. Endogenous peroxidase activity was blocked using a solution of 3% H_2O_2 in methanol (*v*:*v*) for 10 min at room temperature. The slides were then rinsed for 2 × 5 min in 1× PBS pH 7.4 in preparation for heat-induced epitope retrieval. Antigen retrieval was performed for 20 min at 95 °C in either sodium citrate buffer (10 mM sodium citrate, 0.05% Tween 20, pH 6.0) or Tris-EDTA buffer (10 mM Tris Base, 1mM EDTA solution, 0.05% Tween 20, pH 9.0) depending on the primary antibody used (Table S4). The slides were then rinsed for 2 × 5 min in 1× PBS pH 7.4 and blocked in 10% Normal Sheep Serum (NSS) for 1 h at room temperature. Blocking buffer was drained off the slides and primary antibodies were diluted in antibody diluent (0.5% NSS in 1× PBS pH 7.4), as detailed in Table S4, and applied to slides overnight at 4 °C. After the overnight incubation, slides were washed for 2 × 5 min in 1× PBS pH 7.4, and the secondary antibody diluted in antibody diluent (Table S4) was applied to the slides and left to incubate for 1 h at room temperature. Slides were washed again for 2 × 5 min in 1× PBS pH 7.4 before the addition of Diaminobenzidine (DAB) solution (0.05% DAB, 0.015% H_2O_2 in 1× PBS pH 7.4) and the colour was developed for 1–3 min depending on the antibody. The slides were washed for 2 × 5 min in 1× PBS pH 7.4 and counterstained with Haematoxylin Gill 3 (Sigma-Aldrich, St. Louis, MO, USA) for 2 min followed by bluing by rinsing in tap water for 12 min. Finally, the slides were dehydrated through 4 exchanges of alcohol (95%, 95%, 100%, and 100%) for 5 min each and then cleared through 3 changes of 100% Xylene and cover-slipped using Depex mounting media (BDH Biochemicals, London, UK).

Multiplex GeXP Assay

Custom Design of a Multiplex GeXP Assay

Using available online tools (IDT and Life Technologies Oligo Analyzers), all primers in each set were compared to ensure there were no strong intra- or inter-primer interactions, and if there were, primers were redesigned. A forward (5′-AGGTGACACTATAGAATA-3′) and reverse (5′-GTACGACT CACTATAGGGA-3′) universal primer sequence was added the 5′ end of each primer to obtain compatibility with the Genome GeXP Genetic Analysis System (Beckman Coulter, Fullerton, CA, USA). This universal sequence serves as a template for universal primers in the reaction mixtures for use in subsequent amplification steps to ensure equal amplification efficiencies across all genes in a set. All gene PCR products were initially validated in single-plex using whole mouse ovary cDNA as the template, confirming the functionality of the primer pair by the presence of only one expression peak and, therefore, PCR product.

Reverse Transcription (RT)

An RNA template was added to a pool of all reverse primers of varying, pre-optimised concentrations (SI Appendix, Tables S1–S3), reverse transcriptase and associated buffer (as

per the manufacturer's instructions, Beckman Coulter, Fullerton, CA, USA) in a 20 µL reaction volume. A Kanamycin RNA (KanR) exogenous positive control was also included, resulting in a consistent 325 np peak when samples were separated on the GeXP machine. The RT reactions were performed under the following conditions: 1 min at 48 °C, 60 min at 42 °C, and 5 min at 95 °C in a Corbett Rotorgene qPCR machine to avoid the well-to-well variation of a standard thermocycler. The no-template control contained one peak at 325 bp corresponding to the exogenous KanR control and the RT-negative control contained no peaks. In addition to the negative controls, a positive control of an RNA mixture derived from mouse ovary tissue and mouse ovarian cancer cell lines, where all genes are expressed, was included to ensure assay success.

Polymerase Chain Reaction (PCR)

The resulting RT sample was used as the template for the subsequent PCR. Therefore, 9.3 µL of the final RT reaction was mixed with all forward primers of each GeXP set to a concentration of 20 nmol, Thermo-Start DNA Polymerase (Thermo Fisher Scientific, Waltham, MA, USA) and associated GeXP buffer, in a 20 µL final reaction volume (as per the manufacturer's instructions, Beckman Coulter, Fullerton, CA, USA). The PCR was again performed in a Corbett Rotorgene qPCR machine under the following conditions: 10 min at 95 °C, then 35 cycles of 30 s at 94 °C, 30 s at 55 °C and 1 min at 70 °C. PCRs were completed in technical duplicate.

GeXP Sample Separation and Data Analysis

Samples were prepared for analysis following the manufacturer's instructions, which is briefly outlined here. The capillary array was pre-heated to 50 °C for 15 min prior to analysis, followed by 3 × 0.4 mL manifold purges and 3× capillary fill operations. For each sample, 6.15 µL of neat PCR product was added to 1.025 µL of DNA size standard—400 (Beckman Coulter, Fullerton, CA, USA) and 74.825 µL of SLS Buffer (Beckman Coulter, Fullerton, CA, USA). The resulting 82 µL sample was split into 2 × 40 µL samples in the GeXP sample plate with 30 µL of mineral oil overlaid to avoid sample evaporation. Therefore, for every RNA sample, there was a total of four technical replicates. PCR products were separated by the standard fragment analysis method (frag-3 protocol) on the GeXP. Peaks were manually associated with each gene based on size results from previous single-plex optimisation steps and relative fluorescent units were exported to Excel for subsequent analysis. Genes were normalised to the most moderately expressed endogenous control, *Ppia*, in each sample, and the resulting fold-changes of the four technical replicates were averaged. Then, the fold-changes of the biological replicates for each experimental group were averaged for graphical representation.

Supplemental Figures

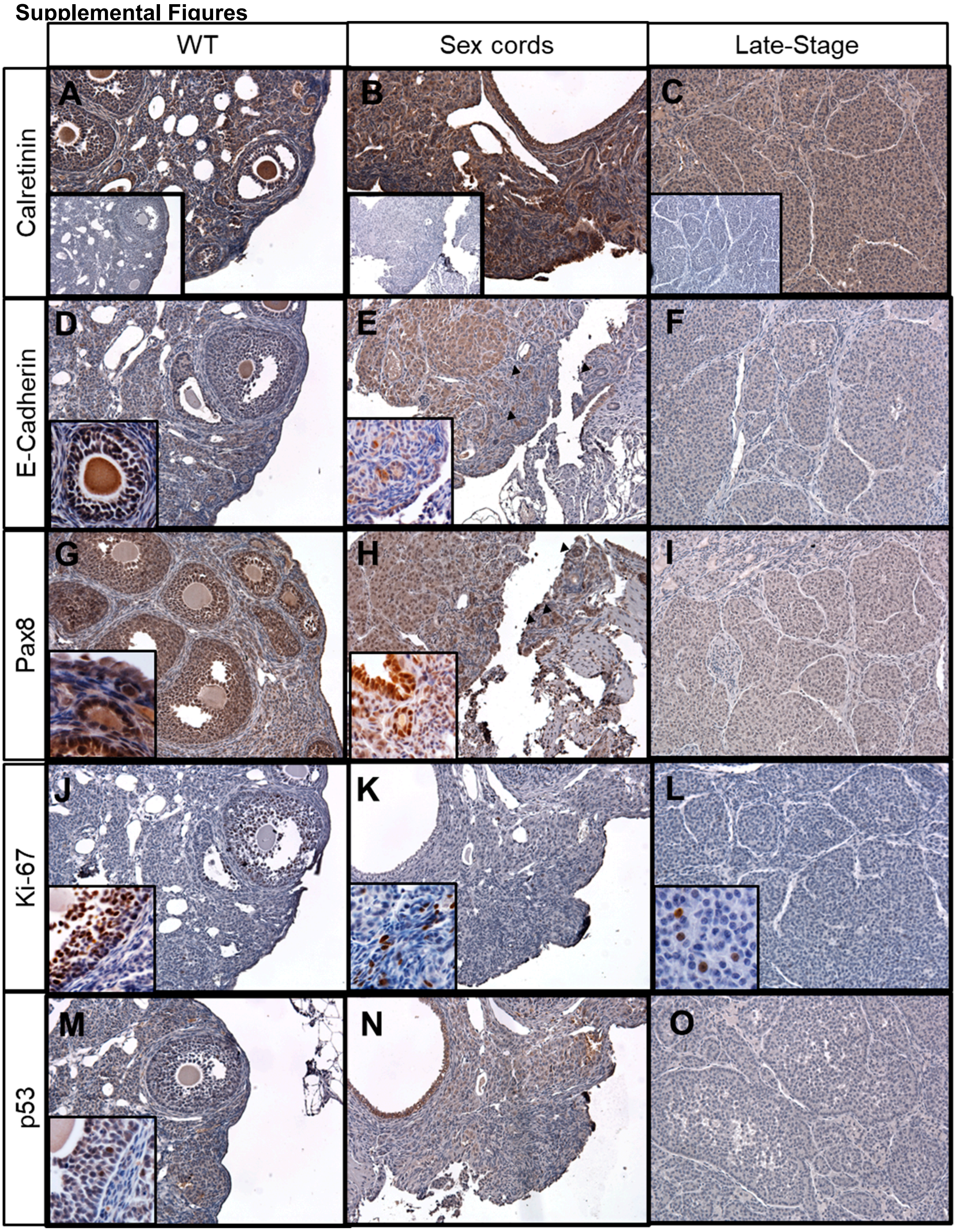

Figure S1: Immunohistochemistry of common epithelial ovarian cancer markers to characterise a late-stage tumour phenotype in *Fancd2*$^{-/-}$ mice. (A,D,G,J,M) depicts ovarian tissue from a 3-month *Fancd2*$^{+/+}$ (wild-type; WT) mouse including primordial, primary and mature follicles, as well as stroma samples. (B,E,H,K,N) depicts ovarian tissue from a 3-month *Fancd2*$^{-/-}$ ovary that includes sex cords (E,H arrowheads) and stroma. (C,F,I,L,O) depicts ovarian tissue from a 1-year *Fancd2*$^{-/-}$ ovary with a later stage ovarian cancer neoplasm. (A,B,C) Calretinin is strongly expressed within all tissues and insets display secondary antibody only controls. (D) E-Cadherin and (G) Pax8 are both present in the ovarian surface epithelium and stroma, with Pax8 more strongly labelled in all granulosa cells, including primary follicles of WT mice. (E) E-Cadherin and (H) Pax8 are present in sex cords and stroma of 3-month *Fancd2*$^{-/-}$ animals, but (F,I) are only present as weak and diffuse staining in the 1-year *Fancd2*$^{-/-}$ phenotype. (J,K,L) Ki-67 staining is limited to a few cells within all samples and (M,N,O) p53 is present, and its weak and diffuse staining suggests normal function. Photomicrographs taken at 20x magnification or 40x for higher magnification in inset images in (D,E,G,H,J,K,L,M).

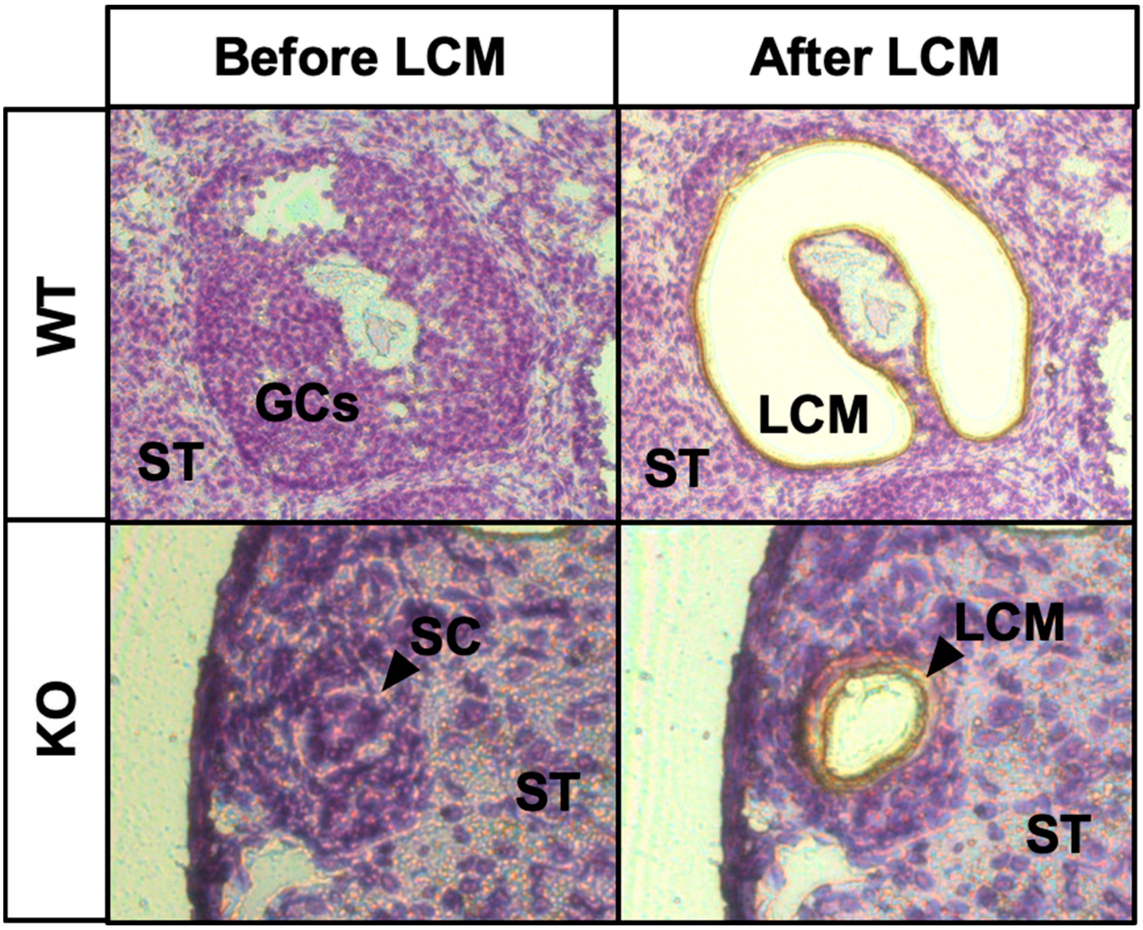

Figure S2: LCM of mature GCs and sex cords. Representative images of tissue before and after laser capture micro-dissection (LCM) performed on *Fancd2*$^{+/+}$ (WT) and *Fancd2*$^{-/-}$ (KO) ovaries to collect mature GCs and sex cords (SC), respectively. ST = ovarian stroma.

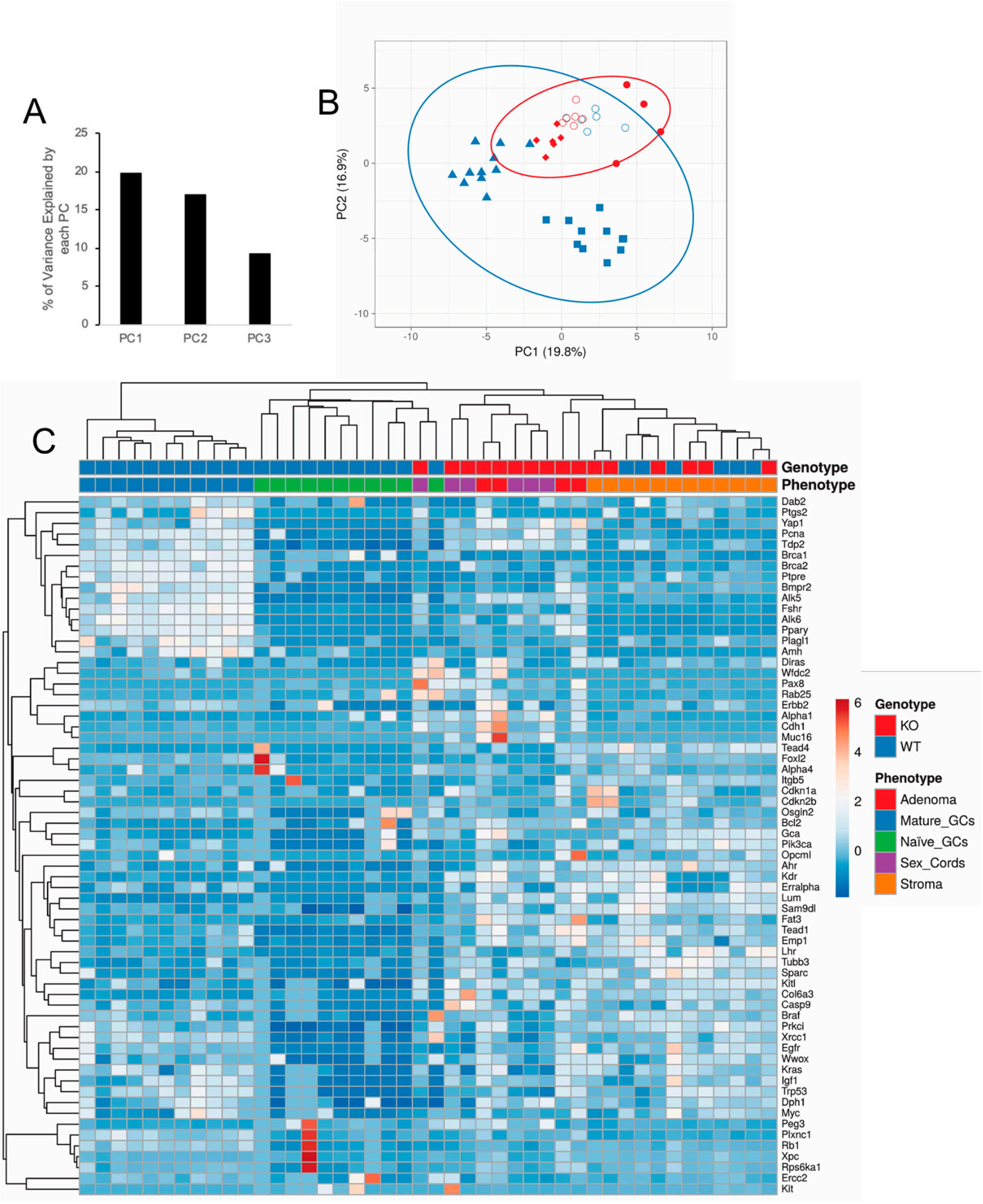

Figure S3: Unbiased analyses of relative gene expression data of sex cords and ovarian stroma from 3-month-old *Fancd2*$^{-/-}$ (KO) mice, tubulostromal adenomas and ovarian stroma from 1-year old KO mice, naïve granulosa cells (GC), mature GC and stroma from 3-month-old *Fancd2*$^{+-+}$ (WT) mice and mature GC and stroma from 1-year old WT mice. (A) Bar graph

displays the percent variance represented by the first three principal components. (B) X and Y axis show principal components 1 and 2, which explain 19.8% and 16.9% of the total variance, respectively. Data segregation based on genotype, WT vs KO, however stromal controls are intermediately mixed. Prediction ellipses are such that with a probability of 0.95, a new observation from the same group will fall inside the ellipse. N = 44 data points (C) Heat map of unsupervised hierarchical clustering with minimal misclassification. Data mirrors results of PCA plot. Rows are centred; unit variance scaling is applied to rows. Imputation is used for missing value estimation. Both rows and columns are clustered using correlation distance and average linkage. 65 rows, 44 columns.

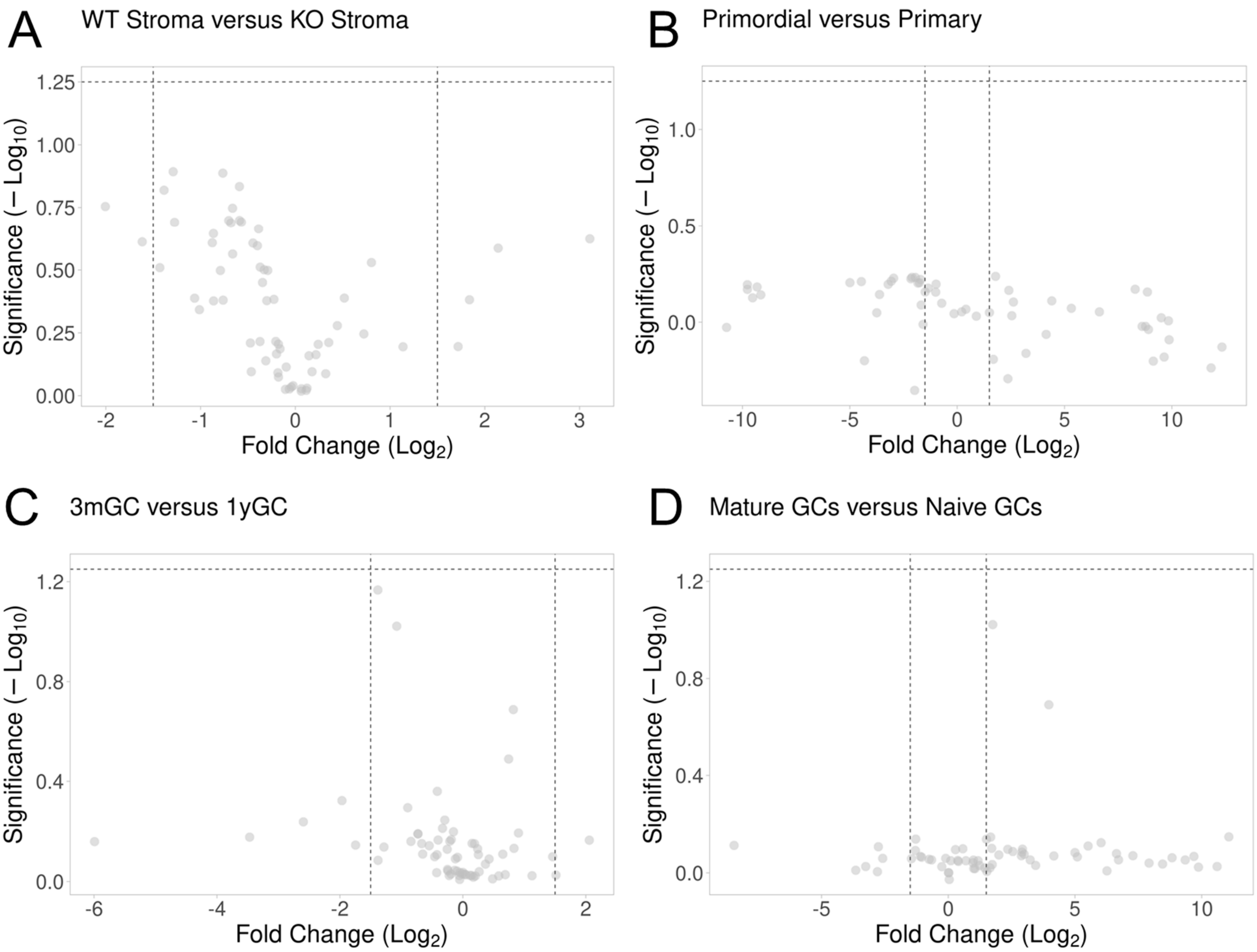

Figure S4: Volcano plots representing non-significant gene expression changes following pairwise comparisons. (A) Ovarian stroma from 3-month-old *Fancd2*$^{+/+}$ (wild type; WT) and *Fancd2*$^{-/-}$ (knock out; KO) mice exhibited similar levels of expression of all genes tested and served as a genotype control. (B) Granulosa cells (GC) from primordial and primary follicles from WT mice exhibited similar levels of expression of all genes tested and confirmed that these sample types could be combined as naïve GCs. (C) GC from growing follicles of 3-month-old and 1-year old WT mice exhibited similar levels of expression of all genes tested and confirmed these sample types could be combined as mature GCs. (D) Naïve and mature GCs from WT mice exhibited similar levels of expression of all genes tested and supports the grouping of these phenotypes for further analyses. Thresholds represented as dashed lines, were set at a fold change of > 1.5 and p-value of < 0.05 for relative gene expression data from all GeXP gene sets. No genes for any of the control comparisons were significant based on designated threshold.

Supplemental Tables

Table S1: Genome GeXP Multiplex Assay Set 1.

Gene Symbol	Accession Number	Primer Sequences (5' – 3')	[Primer μM] F R	Amplicon Length (bp)
Pax8	NM_011040	F- AGGTGACACTATAGAATAAGATCCTTGGCAGGTACT<br>R- GTACGACTCACTATAGGGATGTAGTCTCCTATCTTCTCCA	2<br>0.5	145
Rpl19	NM_009078	F- AGGTGACACTATAGAATACCCACAAGCTCTTTCC<br>R- GTACGACTCACTATAGGGAATCCAACCAGACCTTCTT	2<br>0.5	150
Hprt	NM_013556	F- AGGTGACACTATAGAATACAGGTGTTCTAGTCCTGTG<br>R- GTACGACTCACTATAGGGAGTGCAAATCAAAAGTCTGG	2<br>0.0039	157
Col6α3	NM_001243008	F- AGGTGACACTATAGAATAGCCATTTCTAACTCGTTTCT<br>R- GTACGACTCACTATAGGGATTAGCACCATATTGACAGG	2<br>0.0039	163
Notch3	NM_008716.3	F- AGGTGACACTATAGAATACTCGTATGTACCAAGTAGCC<br>R- GTACGACTCACTATAGGGACTTTCCCAGTGAGTCTACA	2<br>0.5	174
Braf	NM_139294	F- AGGTGACACTATAGAATAGTCTTCCTGCCCAACA<br>R- GTACGACTCACTATAGGGAGTTTCTTCTCTCCATCCTG	2<br>0.0078	182
Dph1	NM_144491	F- AGGTGACACTATAGAATACAACCCTAATATACCTGCTT<br>R- GTACGACTCACTATAGGGAAAGGTTCCCAGAATAAGG	2<br>0.5	187
Erbb2	NM_001003817	F- AGGTGACACTATAGAATAAACTGGTGTGTTCAGATTG<br>R- GTACGACTCACTATAGGGAATTCAGTCTCATCAATGTCC	2<br>0.0078	197
Ccne1	NM_007633	F- AGGTGACACTATAGAATAGAAATCTACCCTCCAAAG<br>R- GTACGACTCACTATAGGGATGGACATAGACATTCAGC	2<br>0.5	202
Brca1	NM_009764	F- AGGTGACACTATAGAATAGACCCCCAAAGAAGTAAT<br>R- GTACGACTCACTATAGGGAACTATCCACTTTCCTCCTG	2<br>0.5	208
Cdkn1a	NM_007669	F- AGGTGACACTATAGAATAAGACATTCAGAGCCACAG<br>R- GTACGACTCACTATAGGGAGACGAAGTCAAAGTTCCAC	2<br>1	219
Casp3	NM_001284409	F- AGGTGACACTATAGAATAGTGCGAGATGAGGTGTT<br>R- GTACGACTCACTATAGGGATCTTAGCGTACCGTTCC	2<br>0.5	224
Myc	NM_001177352	F- AGGTGACACTATAGAATACTCAGTGGTCTTTCCCTAC<br>R- GTACGACTCACTATAGGGATTCTTGCTCTTCTTCAGAGT	2<br>0.5	233
Igf1	NM_010512	F- AGGTGACACTATAGAATATGTGACATTGCTCTAACATC<br>R- GTACGACTCACTATAGGGACAGGTAGAAGAGGTGTGAAG	2<br>0.0039	240

Gene Symbol	Accession Number	Primer Sequences (5’ – 3’)	[Primer μM]<br>F<br>R	Amplicon Length (bp)
Ercc2	NM_010512	F- AGGTGACACTATAGAATAGGAGGTGACCAAACTTATCTA<br>R- GTACGACTCACTATAGGGAGACACTTCCCATCAACATC	2<br>0.5	246
Kit	NM_010512	F- AGGTGACACTATAGAATAACAAAGAGCAAATCCAG<br>R- GTACGACTCACTATAGGGAGGAAACTCCCATTTGTG	2<br>1	257
Dlec1	NM_177117	F- AGGTGACACTATAGAATACTCTCCGTCTGTATAACTCC<br>R- GTACGACTCACTATAGGGAGGGACAGTGTGGCTAGA	2<br>1	263
Aurka	NM_011497	F- AGGTGACACTATAGAATAGAGGGTTTGACTCCTATTTT<br>R- GTACGACTCACTATAGGGAAGGGAACAGTGGTCTTAAC	2<br>0.5	273
Ppia	NM_008907	F- AGGTGACACTATAGAATAGATGACGAGCCCTTG<br>R- GTACGACTCACTATAGGGAGGACCTGTATGCTTTAGGA	2<br>0.5	285
Kitl	NM_013598	F- AGGTGACACTATAGAATAGGTCCCTTAGTAAAAGTGAA<br>R- GTACGACTCACTATAGGGATCCTTAGTAGCACCCTACAT	2<br>0.125	291
Arl11	NM_177337	F- AGGTGACACTATAGAATATGCCCTAACCACTCG<br>R- GTACGACTCACTATAGGGACCCTAGTAAATCCTCCTG	2<br>0.5	299
Bax2	NM_007527	F- AGGTGACACTATAGAATACTACAGGGTTTCATCCAG<br>R- GTACGACTCACTATAGGGAAAAGTAGAAGAGGGCAACC	2<br>0.5	307
Dab2	NM_023118	F- AGGTGACACTATAGAATAAGTCCGTGTACTTTGTGG<br>R- GTACGACTCACTATAGGGACATCATCAATACCGATTAGC	2<br>0.0078	318
Trp53	NM_011640	F- AGGTGACACTATAGAATACCGAAGACTGGATGACT<br>R- GTACGACTCACTATAGGGAAGAAGGGACAAAAGATGAC	2<br>0.5	336
Kras	NM_021284	F- AGGTGACACTATAGAATACCTGTCTCTTGGATATTCTC<br>R- GTACGACTCACTATAGGGAAACACCCTGTCTTGTCTTT	2<br>0.125	345
Diras2	NM_001024474	F- AGGTGACACTATAGAATACCTACCCCAAGTTTGTAAC<br>R- GTACGACTCACTATAGGGAGCTGTAACTGAGCTTTATTG	2<br>0.5	363
Casp9	NM_015733	F- AGGTGACACTATAGAATATCCACTTGAGTTTAGATGG<br>R- GTACGACTCACTATAGGGACCTCTCTCTCCTGTGTGTC	2<br>0.5	377
Brca2	NM_001081001	F- AGGTGACACTATAGAATAGGGTGCTGTGATGTATCTAGT<br>R- GTACGACTCACTATAGGGAGATTCCTCTGTGGTGTTTT	2<br>0.5	383

Table S2: Genome GeXP Multiplex Assay Set 2.

Gene Symbol	Accession Number	Primer Sequences (5' – 3')	[Primer μM] F R	Amplicon Length (bp)
Lum	NM_008524	F- AGGTGACACTATAGAATATTGGCATTAGTCGGTAGT<br>R- GTACGACTCACTATAGGGAGGGGCAGTTACATTCTG	2<br>0.0078	142
Rpl19	NM_009078	F- AGGTGACACTATAGAATACCCACAAGCTCTTTCC<br>R- GTACGACTCACTATAGGGAATCCAACCAGACCTTCTT	2<br>0.5	150
Hprt	NM_013556	F- AGGTGACACTATAGAATACAGGTGTTCTAGTCCTGTG<br>R- GTACGACTCACTATAGGGAGTGCAAATCAAAAGTCTGG	2<br>0.0156	157
Prkci	NM_008857	F- AGGTGACACTATAGAATAGTGAGTTGTGTCAAGTTTGT<br>R- GTACGACTCACTATAGGGACAGGCATCATCAGGTC	2<br>0.0156	169
Peg3	NM_008817	F- AGGTGACACTATAGAATAGTGCGTAGAGTGCTGTG<br>R- GTACGACTCACTATAGGGACTGATCTCCCCTTGCT	2<br>0.5	176
Rb1	NM_009029	F- AGGTGACACTATAGAATAGGCATCTGCATCTTTATC<br>R- GTACGACTCACTATAGGGACATAGCATTATCAACCTTGG	2<br>0.5	184
Samd9l	NM_010156	F- AGGTGACACTATAGAATACGAAACTGGAAACTGAAG<br>R- GTACGACTCACTATAGGGAGTCCGTGACAAACAACTT	2<br>0.5	196
Rab25	NM_016899	F- AGGTGACACTATAGAATATATCTCCACCTCCCTTACT<br>R- GTACGACTCACTATAGGGACCAGGCACAATACAAAAC	2<br>0.5	206
Tubb3	NM_023279	F- AGGTGACACTATAGAATACAAGACAAGCAGCATCT<br>R-GTACGACTCACTATAGGGAATACAGAGGTGGCTAAAATG	2<br>0.5	212
Bcl2	NM_009741	F- AGGTGACACTATAGAATAGATGCCTTTGTGGAACT<br>R- GTACGACTCACTATAGGGACTGCTTTAGTGAACCTTTTG	2<br>0.5	221
Kdr	NM_010612	F- AGGTGACACTATAGAATATATGAGCCAAGTGTTAAGTG<br>R- GTACGACTCACTATAGGGAGTAACCCGACTGTGAAGA	2<br>0.5	230
Osgin2	NM_145950	F- AGGTGACACTATAGAATAGTTTGGGTATTGGCAGA<br>R- GTACGACTCACTATAGGGAGCAGTAACACATTTCACAG	2<br>0.5	236
Gca	NM_145523	F- AGGTGACACTATAGAATAGATACCTGGGTTATTCTGAC<br>R- GTACGACTCACTATAGGGATTTCCTGTGTAGTCTCTATCC	2<br>0.0156	245
Xpc	NM_009531	F- AGGTGACACTATAGAATAGGCTGTGTCCAGATGAC<br>R- GTACGACTCACTATAGGGACCTTTTTCTCCTTCTCCT	2<br>0.5	251

Gene Symbol	Accession Number	Primer Sequences (5' – 3')	[Primer μM] F R	Amplicon Length (bp)
Ptpre	NM_001316678	F- AGGTGACACTATAGAATAATCCTTCATAGATGGCTAC<br>R- GTACGACTCACTATAGGGAGGTGTCGCTCTTTATCTCT	2<br>0.5	263
Itgb5	NM_001145884	F- AGGTGACACTATAGAATATACAAAACTGCTAAGGACTG<br>R- GTACGACTCACTATAGGGACGCTCACTTTGGAACT	2<br>0.0156	270
Sparc	NM_009242	F- AGGTGACACTATAGAATACGAGACTTTGAGAAGAACTAC<br>R- GTACGACTCACTATAGGGAGATGTCCTGCTCCTTGAT	2<br>0.0156	277
Ppia	NM_008907	F- AGGTGACACTATAGAATAGATGACGAGCCCTTG<br>R- GTACGACTCACTATAGGGAGGACCTGTATGCTTTAGGA	2<br>0.5	285
Plxnc1	NM_018797	F- AGGTGACACTATAGAATAGTTTGCATCCAGTTTGAC<br>R- GTACGACTCACTATAGGGAAATTAGGAGCTAGAAACCTG	2<br>0.5	290
Xrcc1	NM_009532	F- AGGTGACACTATAGAATATTTGTGGAGGTGCTAGTG<br>R- GTACGACTCACTATAGGGAAGGAGGGCTGTGAAACT	2<br>0.5	304
Egfr	NM_207655	F- AGGTGACACTATAGAATATGTGCCCACTACATTGA<br>R- GTACGACTCACTATAGGGAACGTCTTCGCATGAATAG	2<br>1	313
Cdkn2b	NM_007670	F- AGGTGACACTATAGAATAAACCTGGAGAGTAGATGAGA<br>R- GTACGACTCACTATAGGGAAGAGCAACTCAAATGTAGGA	2<br>0.0156	331
Pik3ca	NM_008839	F- AGGTGACACTATAGAATAGAGGAGAACCCTTATGTGA<br>R- GTACGACTCACTATAGGGATGACCCAGTAACACCAATA	2<br>0.0156	342
Opcml	NM_177906	F- AGGTGACACTATAGAATACCATCTCATAGTGCAAGTTC<br>R- GTACGACTCACTATAGGGACCAGTGTTCTTGGCTTT	2<br>0.5	349
Plagl1	NM_009538	F- AGGTGACACTATAGAATACTGCTCTCACAGAAGATACC<br>R- GTACGACTCACTATAGGGAGCTTATACTTGGAGACGAAG	2<br>0.5	358
Rps5ka1	NM_009097	F- AGGTGACACTATAGAATACGACACCTTCTACTTTGATA<br>R- GTACGACTCACTATAGGGAGGATCTCTTTTGCTCTTGT	2<br>0.5	367
Wwox	NM_019573	F- AGGTGACACTATAGAATAGCTGCTACCACTGTCTACTG<br>R- GTACGACTCACTATAGGGATCCCTGCTTCCCACTA	2<br>0.5	372
Rassf1	NM_001243748	F- AGGTGACACTATAGAATAAGCCAGAACTCATTGAACTAC<br>R- GTACGACTCACTATAGGGATCGTATCCCGCTCTAGT	2<br>0.5	379
Akt2	NM_001110208	F- AGGTGACACTATAGAATATCCCTTGGTCTACTACGTC<br>R- GTACGACTCACTATAGGGAGCTGTCACCTAGCTTGAG	2<br>0.5	387

Table S3: Genome GeXP Multiplex Assay Set 3.

Gene Symbol	Accession Number	Primer Sequences (5' – 3')	[Primer µM] F R	Amplicon Length (bp)
Lhr	NM_013582.2	F- AGGTGACACTATAGAATAGTGAAAGCACAGTTAGAGAA<br>R- GTACGACTCACTATAGGGATGGGTGAACAGAAGTCATA	2<br>0.002	142
Rpl19	NM_009078	F- AGGTGACACTATAGAATACCCACAAGCTCTTTCC<br>R- GTACGACTCACTATAGGGAATCCAACCAGACCTTCTT	2<br>0.5	150
Hprt	NM_013556	F- AGGTGACACTATAGAATACAGGTGTTCTAGTCCTGTG<br>R- GTACGACTCACTATAGGGAGTGCAAATCAAAAGTCTGG	2<br>0.0039	157
Ahr	NM_013464.4	F-AGGTGACACTATAGAATATCTGGTTTTCTGGCAAT<br>R- GTACGACTCACTATAGGGAGGCTGAAGTGGAGTAGC	2<br>0.0015625	165
Pcna	NM_011045.2	F- AGGTGACACTATAGAATAGAAGCACCAAATCAAGAG<br>R- GTACGACTCACTATAGGGAGCTAAGGTCTCGGCATA	2<br>0.0039	184
Pgc1α1	NM_008904.2	F- AGGTGACACTATAGAATAGGACATGTGCAGCCAAGACTCT<br>R- GTACGACTCACTATAGGGACACTTCAATCCACCCAGAAAGCT	2<br>0.5	193
Foxl2	NM_012020.2	F- AGGTGACACTATAGAATAGCCCAGATGACACTCCTA<br>R- GTACGACTCACTATAGGGACGAAAGACATTACTCAACCA	2<br>0.0039	201
Pgc1α4	NR_132764.1	F- AGGTGACACTATAGAATATCACACCAAACCCACAGAAA<br>R- GTACGACTCACTATAGGGACTGGAAGATATGGCACAT	2<br>0.5	209
Alk6	NM_007560.4	F- AGGTGACACTATAGAATATGTCGTGTTGTCTAAATCTC<br>R- GTACGACTCACTATAGGGATGTAAGAGGTCCATACCAG	2<br>0.5	216
Tead1	NM_001166584.1	F- AGGTGACACTATAGAATAGCTGAAGGTAACAAGCA<br>R- GTACGACTCACTATAGGGATGGCTGTCCTGTCTGTAT	2<br>0.5	224
Cdh1	NM_009864.3	F- AGGTGACACTATAGAATATCTCTTGTCCCTTCCAC<br>R- GTACGACTCACTATAGGGAAGTGTCCCTCCAAATCC	2<br>0.5	232
Tdp2	NM_019551.2	F- AGGTGACACTATAGAATAGCACCTTATTCCTCAAAGT<br>R- GTACGACTCACTATAGGGATGTATTAGACCCAGATGTCC	2<br>0.125	237
Fat3	NM_001080814.1	F- AGGTGACACTATAGAATATGTCCTCAACATAACCATC<br>R- GTACGACTCACTATAGGGAATTACCTCACCATTAGAACC	2<br>0.5	247
Emp1	NM_010128.4	F- AGGTGACACTATAGAATAATCTCCCTTGTGGTCTTC<br>R- GTACGACTCACTATAGGGAAAGCTGAAACAGAAGCAG	2<br>0.5	252

Gene Symbol	Accession Number	Primer Sequences (5' – 3')	[Primer μM] F R	Amplicon Length (bp)
Muc16	XM_011242634.1	F- AGGTGACACTATAGAATACCAGAGTCCAACACAGTTAT<br>R- GTACGACTCACTATAGGGAGTGAACATCCCATTCTTCT	2<br>0.125	258
Pparγ	NM_001127330.2	F- AGGTGACACTATAGAATATCACCATTTGTCATCTACG<br>R- GTACGACTCACTATAGGGAACACCATACTTGAGCAGAG	2<br>0.5	270
Bmpr2	NM_007561.4	F- AGGTGACACTATAGAATAAATCAGACGAAGAGCAG<br>R- GTACGACTCACTATAGGGATCTCACCTATCCCAAGAT	2<br>0.0039	279
Ppia	NM_008907	F- AGGTGACACTATAGAATAGATGACGAGCCCTTG<br>R- GTACGACTCACTATAGGGAGGACCTGTATGCTTTAGGA	2<br>0.125	285
Ptgs2	NM_011198.4	F- AGGTGACACTATAGAATAGGAACTAACCAAGAACAAAG<br>R- GTACGACTCACTATAGGGAATGGGCTGGAAGACATA	2<br>0.5	295
Tead4	NM_011567.2	F- AGGTGACACTATAGAATAAAGATGTATGGTCGGAATG<br>R- GTACGACTCACTATAGGGAAAACCCTGAGATTGCTG	2<br>1	307
Alk5	NM_009370.3	F- AGGTGACACTATAGAATAGCTTAGTGTTCTGGGAAAT<br>R- GTACGACTCACTATAGGGACTGTTGGCTGAGTTGTG	2<br>0.125	318
Fshr	NM_013523.3	F- AGGTGACACTATAGAATACGTCAAGAGACACCAAGAT<br>R- GTACGACTCACTATAGGGATTGTGGGTAATAGATGAGGT	2<br>0.125	338
Errα	NM_007953.2	F- AGGTGACACTATAGAATAAAAGTGAATGCCCAGGT<br>R- GTACGACTCACTATAGGGACTGCTTGGAGTTATTGCTT	2<br>1	343
Mmp9	NM_013599.4	F- AGGTGACACTATAGAATAACAGCACCTCCCACTAT<br>R- GTACGACTCACTATAGGGAGGGCTTCCTCTATGATT	2<br>1	348
Fcgbp	NM_001122603.1	F- AGGTGACACTATAGAATACCTGACAACAGCCACTAT<br>R- GTACGACTCACTATAGGGACAAGGCACACTCCTCAC	2<br>0.5	355
Yap1	NM_001171147.1	F- AGGTGACACTATAGAATACTTGGTTATCTCCTCTCTACA<br>R- GTACGACTCACTATAGGGAAATCAGGCTAAGGGAAGTA	2<br>0.5	360
Taz	NM_001173547.2	F- AGGTGACACTATAGAATACTGAAGTTGATGCGTTG<br>R- GTACGACTCACTATAGGGAAGTAGGGTGGGCTGTTAG	2<br>0.5	365
Wfdc2	NM_026323.2	F- AGGTGACACTATAGAATAGCTCTCAGGGACAGATACT<br>R- GTACGACTCACTATAGGGAGGCAGAAACAACCTCTCT	2<br>0.5	370
Amh	NM_007445.2	F- AGGTGACACTATAGAATAAGGGGAGACTGGAGAAC<br>R- GTACGACTCACTATAGGGATCAGAGCCAAATAGAAAGG	2<br>0.5	385

Table S4: Detailed information about antibodies used for immunohistochemistry.

Supplier	Catalogue Number	Target	Dilution	Antigen Retrieval Solution
**Abcam**	ab92341	Calretinin	1:100	Sodium Citrate
**Invitrogen**	PA585088	E-Cadherin	1:500	Sodium Citrate
**Abcam**	ab97477	Pax8	1:500	Tris-EDTA
**Abcam**	ab16667	Ki-67	1:200	Sodium Citrate
**Abcam**	ab131442	p53	1:200	Sodium Citrate
**Abcam**	ab6721	Goat α Rabbit IgG HRP	1:300	N/A

Table S5: Summary of staining intensity of immunohistochemical markers in representative samples. Three-month wild-type (WT) samples represents staining of granulosa cells within primordial, primary and growing follicles as well as stroma. Sex cords represents both sex cord structures and stroma within 3-month old *Fancd2*$^{-/-}$ mice and late-stage represents adenomas from 1-year old *Fancd2*$^{-/-}$ mice.

Marker	3-month WT	Sex Cords	Late-Stage
**Calretinin**	+++	+++	+++
**E-Cadherin**	+	++	+
**Pax8**	++	++	++
**Ki-67**	++	+	+
**p53**	++	++	++

Table S6: Summary of all pairwise comparisons made for Gene Set Enrichment Analysis and the resulting number of enriched gene ontology (GO) terms for each comparison.

		Compared to:			
		Naïve GCs	Mature GCs	Sex Cords	Adenoma
GO Terms Enriched in:	Naïve GCs		0	0	0
	Mature GCs	20		10	8
	Sex Cords	23	0		1 p=0.053
	Adenoma	0	0	0	
